# Supplementary material for: The Design of Rapid Self-Healing Alginate Hydrogel with Dendritic Crosslinking Network
Source: Molecules. 2022 Oct 29;27(21):7367. doi: 10.3390/molecules27217367 (PMC9655740; doi:10.3390/molecules27217367)
Supplement: Supplementary file 1 [file molecules-27-07367-s001.zip › molecules-1928631-supplementary.pdf]

Supplementary Materials

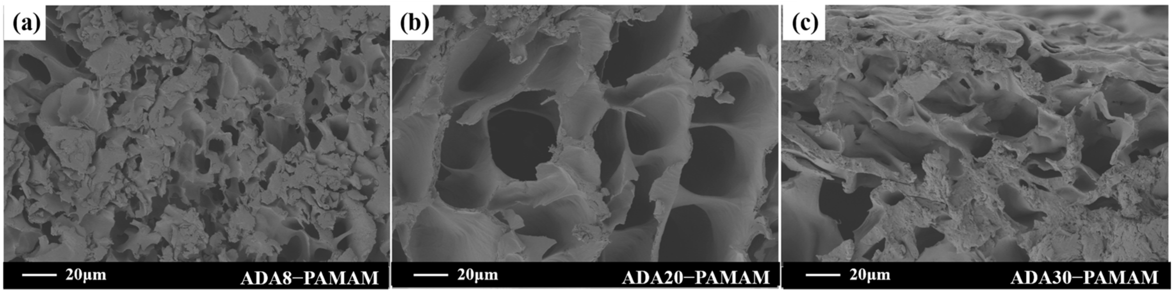

Figure S1. Network morphology of ADA-PAMAM with different oxidation degrees.

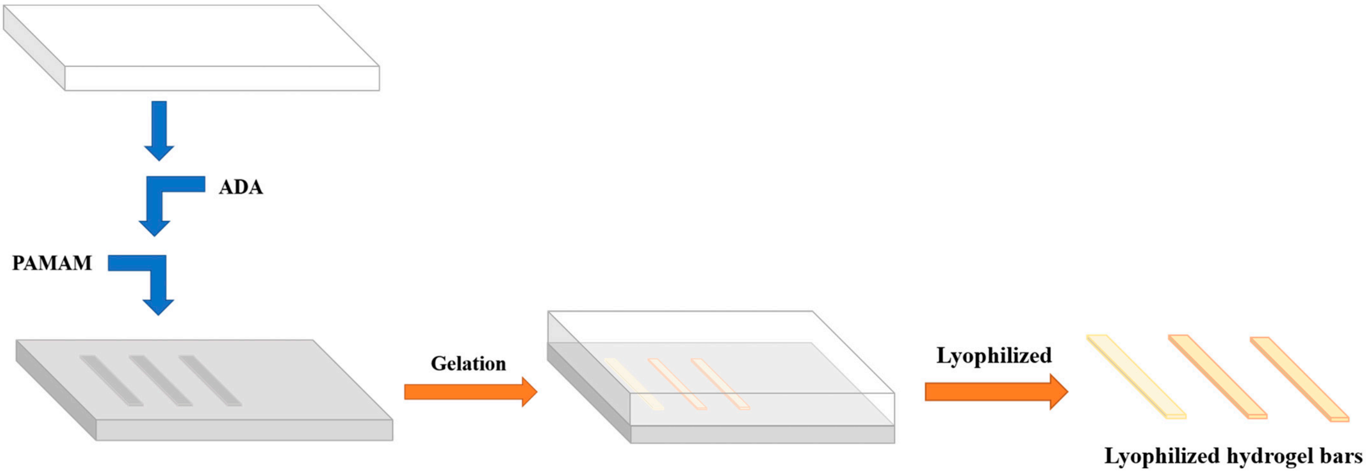

Figure S2. Synthesis of hydrogel lyophilized bars.

Table S1. Weight average molecular weight (Mw) and aldehyde degree of ADA with different oxidation degree.

Oxidation degree [a] (%)	Mw [b] (kDa)	Aldehyde degree [c] (%)
0	121±15.2	—
8	67±6.3	7.0±0.9
20	43±4.1	16.3±2.2
30	39±3.7	25.2±2.5

[a] Oxidation degree is the weight ratio between sodium periodate and the monomer of sodium alginate before the oxidation reaction.

[b] The Mw was calculated based on the refractive index signal, multiple angles light scattering signal and the dn/dc value of sodium alginate.

[c] Aldehyde degree is the molar ratio between free aldehyde groups on the chains and the monomer of sodium alginate, which was calculated by Beer-Lambert Law.
